# Characterization of the Fecal Microbiota Using High-Throughput Sequencing Reveals a Stable Microbial Community during Storage

**DOI:** 10.1371/journal.pone.0046953

**Published:** 2012-10-05

**Authors:** Ian M. Carroll, Tamar Ringel-Kulka, Jennica P. Siddle, Todd R. Klaenhammer, Yehuda Ringel

**Affiliations:** 1 Division of Gastroenterology and Hepatology, Department of Medicine, University of North Carolina at Chapel Hill, Chapel Hill, North Carolina, United States of America; 2 Gillings School of Global Public Health, University of North Carolina at Chapel Hill, Chapel Hill, North Carolina, United States of America; 3 Department of Food Science, North Carolina State University, Raleigh, North Carolina, United States of America; University of Waterloo, Canada

## Abstract

The handling and treatment of biological samples is critical when characterizing the composition of the intestinal microbiota between different ecological niches or diseases. Specifically, exposure of fecal samples to room temperature or long term storage in deep freezing conditions may alter the composition of the microbiota. Thus, we stored fecal samples at room temperature and monitored the stability of the microbiota over twenty four hours. We also investigated the stability of the microbiota in fecal samples during a six month storage period at −80°C. As the stability of the fecal microbiota may be affected by intestinal disease, we analyzed two healthy controls and two patients with irritable bowel syndrome (IBS). We used high-throughput pyrosequencing of the 16S rRNA gene to characterize the microbiota in fecal samples stored at room temperature or −80°C at six and seven time points, respectively. The composition of microbial communities in IBS patients and healthy controls were determined and compared using the Quantitative Insights Into Microbial Ecology (QIIME) pipeline. The composition of the microbiota in fecal samples stored for different lengths of time at room temperature or −80°C clustered strongly based on the host each sample originated from. Our data demonstrates that fecal samples exposed to room or deep freezing temperatures for up to twenty four hours and six months, respectively, exhibit a microbial composition and diversity that shares more identity with its host of origin than any other sample.

## Introduction

The intestinal microbiota is a complex community of bacteria, archaea, and eukarya. The bacterial fraction is believed to contain more than 500 different species and can reach numbers of 10^12^–10^14^ cells/ml of luminal contents [1], [2], with the highest densities residing in the colon. Given this complex microbial community’s association with disease [3], [4], accurate characterization of the composition and diversity of the intestinal microbiota within biological samples is of the utmost importance.

Between 40–60% of the bacteria residing within the gut are reported to be un-culturable [5]. Thus, current research relies upon DNA-based culture-independent methods for characterization of the intestinal microbiota [6], [7]. The handling and treatment of biological samples used to characterize the intestinal microbiota is critical when comparing the composition of this complex microbial community between different ecological niches or diseases. This is particularly important in studies investigating the association of the intestinal microbiota with irritable bowel syndrome (IBS), where fecal samples have been left at room temperature for up to six hours before isolation of fecal DNA [8]. A recent report highlighted the significant effect of ‘length of time’ at ‘room temperature’ (approximately 25°C) on the composition of the microbiota in a cystic fibrosis sputum sample, whereas freezing temperatures (−20 and −80°C) did not significantly alter the composition of the microbiota [9]. Additionally, the effect of a range of temperatures (20, 4, −20, and −80°C) at two time points (3 and 14 days) on the fecal microbiota has been assessed [10]. This study reported a lack of effect of time and temperature on the composition of the microbiota in fecal samples. However, this study compared the fecal microbiota at three and fourteen days, but did not include a baseline sample that is reflective of the fecal microbiota at the time of defecation. Thus, the aim of our study was to characterize the composition of the microbiota in fecal samples left at room temperature at baseline (30 minutes following defecation) and monitor the stability of the microbiota at six time points over twenty four hours. Additionally, we sought to investigate the stability of the microbiota in fecal samples at seven time points during a six month storage period at −80°C. As the fecal microbiota in healthy individuals and patients with intestinal disease may be affected differently by the length of time at a given temperature, we included IBS patients and healthy controls in our study.

## Materials and Methods

### Ethics Statement

The study was approved by the UNC Internal Review Board (IRB) and all subjects provided written consent prior to participation in the study.

### Study Population

We studied 2 patients that met the Rome III criteria for IBS and 2 healthy controls. Subjects were recruited from the Chapel Hill general population and from the University of North Carolina (UNC) healthcare outpatient clinics by advertising.

Inclusion criteria for all subjects were as previously described [11]. Briefly, subjects 18 years or older, and of any gender, race, or ethnicity were recruited for this study. Healthy controls had no recurring GI symptoms. IBS subjects with a history of GI tract surgery (other than appendectomy or cholecystectomy), a history of inflammatory bowel diseases (IBD), celiac disease, lactose malabsorption, or any other diagnosis that could explain chronic or recurring bowel symptoms were excluded from the study. In addition, participants were excluded if they had a history of treatment with antibiotics, anti-inflammatory agents including aspirin, non-steroid anti-inflammatory drugs (NSAIDs or steroids), or if they had intentionally consumed probiotics two months prior to the study. An eight-week wash-out period was required for subjects who reported intentional consumption of probiotics prior to enrollment. All subjects were evaluated by a physician to exclude an alternative diagnosis to IBS.

### Sample Collection and Preparation

**Figure 1:**
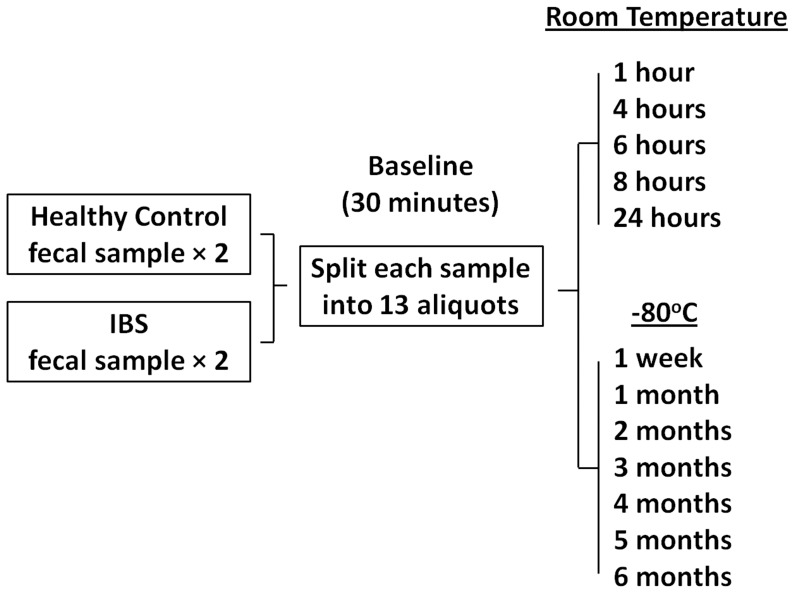
Schematic of experimental design.

The strategy for our study is outlined in Figure 1. A fresh stool sample was collected from all subjects (*n* = 4) on site and immediately transported to the laboratory where it was mechanically homogenized with a sterile spatula. Each sample was split into thirteen aliquots. Each aliquot contained 200 mg of stool in a sterile 2 ml cryovial. Fecal aliquots were then either stored at (I) room temperature (approximately 25°C) or (II) −80°C for different lengths of time. ***Room temperature***: at thirty minutes following defecation a baseline sample aliquot was transferred to −80°C. Five sample aliquots were then sequentially transferred to −80°C following 1, 4, 6, 8 and 24 hours exposure to room temperature. Total bacterial DNA was then isolated from all seven room temperature sample aliquots for each subject. −80°C: at baseline seven sample aliquots for each subject was transferred to −80°C. Following 1 week at −80°C one sample aliquot for each subject was removed from storage and total bacterial DNA was isolated. At 1, 2, 3, 4, 5 and 6 months at −80°C a sample aliquot for each subject was removed from storage and total bacterial DNA was isolated. Total bacterial DNA from each sample aliquot for each subject was used for characterization of the intestinal microbiota.

### Isolation of DNA

Bacterial DNA was isolated from a total of 52 fecal sample aliquots (4 subjects×13 time points) using a phenol/chloroform extraction method combined with physical disruption of bacterial cells and a DNA clean-up kit (Qiagen DNeasy® Blood and Tissue extraction kit [Qiagen, Valencia, CA]) as previously described[11]–[13]. Briefly, 100 mg of frozen feces was suspended in 750 µl of sterile bacterial lysis buffer (200 mM NaCl, 100 mM Tris [pH 8.0], 20 mM EDTA, 20 mg mL^−1^ lysozyme) and incubated at 37°C for 30 min. Next, 40 µl of proteinase K (20 mg mL^−1^) and 85 µl of 10% SDS was added to the mixture and incubated at 65°C for 30 min. 300 mg of 0.1 mm zirconium beads (BioSpec Products, Bartlesville, OK) was then added and the mixture was homogenized in a bead beater (TeSeE process 48®, Bertin Technologies, Le Bretonneux, France) for 90 seconds at 5300 rpm. The homogenized mixture was cooled on ice and then centrifuged at 14,000 rpm for 5 min (Eppendorf centrifuge 5417R, rotor F45-30-11). The supernatant was transferred to a new 1.5 ml microfuge tube and fecal DNA was further extracted by phenol/chloroform/iso-amyl alcohol (25∶24:1) and then chloroform/iso-amyl alcohol (24∶1). Following extraction the supernatant was precipitated by absolute ethanol at −20°C for 1 hour. The precipitated DNA was suspended in DNase free H_2_O and then cleaned using the DNeasy® Blood and Tissue extraction kit (Qiagen, Valencia, CA) from step 3 as per the manufacturer’s instructions. Prior to microbial characterization, isolated fecal DNA was stored at −80°C until DNA was obtained from all samples.

### 454 Pyrosequencing of 16S rRNA Genes

Bacterial community composition in isolated DNA samples was characterized as previously described [14]. Briefly, the V1-3 (forward, 8f:5′-AGAGTTTGATCMTGGCTCAG-3′; reverse 518r: 5′-ATTACCGCGGCTGCTGG-3′) variable region of the 16S rRNA gene was amplified by polymerase chain reaction (PCR). Forward primers were tagged with 10 bp unique barcode labels at the 5′ end along with the adaptor sequence (5′-CCATCTCATCCCTGCGTGTCTCCGACTCAG-3′) to allow multiple samples to be included in a single 454 GS FLX Titanium sequencing plate as previously described [15]. 16S rRNA PCR products were quantified, pooled, and purified for the sequencing reaction. 454 GS FLX Titanium sequencing was performed on a 454 Life Sciences Genome Sequencer FLX machine (Roche, Florence, SC) at the microbiome core at UNC-Chapel Hill (http://www.med.unc.edu/microbiome).

### Analysis of 16S rRNA Sequences using the QIIME Pipeline

16S rRNA sequence data generated by the 454 GS FLX Titanium sequencer was processed by the quantitative insights into microbial ecology (QIIME) pipeline [16]. Briefly, sequences that were less than 200 bp or greater than 1,000 bp in length, contained incorrect primer sequences, or contained more than 1 ambiguous base were discarded. The remaining sequences were assigned sample aliquots based on their unique nucleotide barcodes, including error-correction [17]. Chimeric sequences were removed using ChimeraSlayer [18]. Sequences were clustered into Operational Taxonomic Units (OTUs) based on their sequence similarity at 97% sequence similarity (similar to species level) using UCLUST [19]. A representative sequence for each OTU was chosen for downstream analysis based on the most abundant sequence from each OTU. PyNAST was used to align sequences with a minimum length of 150 bp and a minimum percent identity of 75.0 [20]. OTUs were assigned to a taxonomy using the Ribosomal Database Project (RDP) Naive Bayes classifier v 2.2 with the confidence level set at 0.8 [21]. β-diversity (diversity between groups of samples) was used to generate principal coordinate plots for each sample using un-weighted and weighted UniFrac distances[22]–[24]. PCoA plots were used to visualize the similarities or dissimilarities of variables that best represent the pair-wise distances between sample groups.

### Statistical Analysis

Before statistical comparisons were made bacterial taxa percentages and average UniFrac value data sets were assessed for normality using the D’Agostino and Pearson omnibus normality test. When a data set was identified as not having a normal distribution it was transformed by log_10_ [25], and retested for normality. Data sets normally distributed were compared using a Student’s t-test. All statistical comparisons were carried out using GraphPad software (v4.0a; Prism, San Diego, CA). We used taxon and phylogenetic-based analyses to compare 16S rRNA gene sequences within IBS patients and healthy controls over time at room temperature or −80°C. Taxon-based: The means and standard deviations of abundances of bacterial groups (Phylum, Class, Order, Family, and Genus) and OTUs were calculated and compared between all samples stored at room temperature and −80°C, and between samples stored at room temperature and −80°C for each individual. A *p* value of less than 0.05 was considered significant after testing for multiple comparisons using Bonferroni correction. Zero values for taxa and OTUs were considered as absences and not included when calculating averages. Phylogenetic-based: Phylogenetic trees for IBS patients and healthy controls were generated using the QIIME pipeline [16]. Each tree was subjected to weighted and unweighted UniFrac analysis[22]–[24] through the QIIME pipeline. UniFrac distances represent the fraction of branch length that is shared by any two samples’ communities in a phylogenetic tree built from 16S rRNA sequence data from all samples. Statistical differences between groups of samples (i.e. room temperature versus −80°C samples, and room temperature and −80°C samples from each individual) were tested using analysis of similarity (ANOSIM – available through QIIME) by permutation of group membership with 999 replicates. The test statistic R, which measures the strength of the results, ranges from −1 to 1: R = 1 signifies differences between groups, while R = 0 signifies that the groups are identical.

## Results

### I. Characterization of Microbial Communities in IBS and HC Fecal Samples

A total of 201,101 16S rRNA sequences with acceptable quality were obtained from 52 fecal samples (4 subjects×13 aliquots) with an average of 3,867 reads (range: 1,552–6,531) per sample (HC: 4,309 reads per sample, on average, with a range of 1,936–6,531; IBS: 3,426 reads per sample, on average, with a range of 1,552–5,797). 18.14% of the total number of sequences were found to be chimeric and removed from further analyses. In order to determine the number and abundances of different bacterial groups in each sample we used 3% dissimilarity between 16S rRNA gene sequences as an indicator of a ‘species level’ OTU. Using this procedure, we found a total of 8,334 OTUs in all samples obtained from the 4 subjects. The 16S rRNA sequence data for this study is publically available at the QIIME website (www.microbio.me/qiime) under the title ‘storage study’.

### II. Stability of Fecal Microbial Communities During Storage

**Figure 2:**
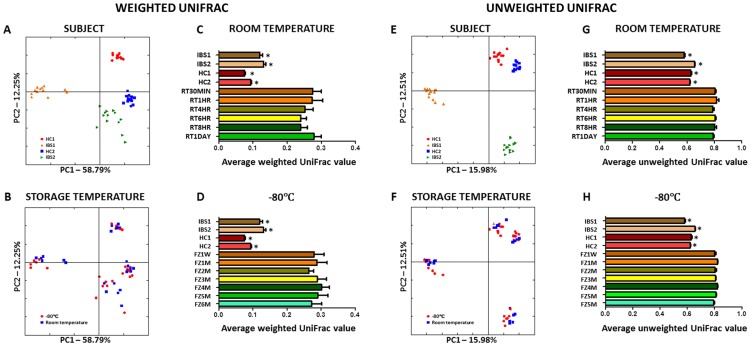
β-diversity analysis of samples. Principal coordinates analysis (PCoA) of weighted and unweighted UniFrac distances of IBS patient (green and orange triangles) and healthy control (blue squares and red circles) fecal sample aliquots exposed to room temperature and −80°C for different lengths of time (room temperature - 1, 4, 6, 8 and 24 hours; −80°C −1 week and 1, 2, 3, 4, 5 and 6 months). PCoA plots illustrate the subject each sample aliquot originated from (**A&E**) and the temperature they were stored at (**B&F**). Average weighted UniFrac distances for all sample aliquots based on storage at room temperature (**C&G**) or −80°C (**D&H**) indicate that sample aliquot microbiotas show significantly similarity (*p<0.05).

To compare the global composition of the microbiota in HC and IBS fecal samples stored at room temperature and −80°C for different lengths of time UniFrac distances[22]–[24] were calculated and compared between sample aliquots. Principal Coordinate Analysis (PCoA) of weighted and unweighted UniFrac distances for all sample aliquots stored at room temperature or −80°C (*n* = 52) revealed four distinct clusters (Figure 2 A&E). Each cluster contained samples obtained from one subject for all time points at both room temperature and −80°C. Additionally, each cluster contained samples obtained from one subject for all time points. PCoA plots of unweighted UniFrac distances revealed no significant clustering of samples based on storage temperature. PCoA plots of weighted UniFrac distances revealed no significant clustering of samples based on storage temperature for both HC samples and one IBS sample, however samples from one IBS individual (IBS1) exhibited clustering based on storage temperature (Figure 2 B&F). ANOSIM confirmed that the samples from the two HC and one IBS individual did not cluster based on storage temperature (HC1: unweighted Unifrac - R = 0.16 *p* = 0.07, weighted UniFrac - R = 0.01 *p* = 0.39; HC2: unweighted Unifrac - R = 0.07 *p* = 0.72, weighted UniFrac - R = 0.07 *p* = 0.22; IBS2: unweighted Unifrac - R = −0.05 *p* = 0.70, weighted UniFrac - R = 0.14 *p* = 0.08), and that samples from one IBS individual did cluster based on storage temperature (IBS1: unweighted Unifrac - R = −0.01 *p* = 0.48, weighted UniFrac - R = 0.21 *p* = 0.04). Average weighted and unweighted UniFrac distances calculated based on the ‘host of origin’ were found to be significantly lower (*p*<0.05) when compared to UniFrac distances calculated based on the length of time a sample was stored at room temperature or −80°C (Figure 2 C,D,G&H), indicating that the microbiota in fecal samples are more similar within the individual of origin when compared to the length of time at a storage temperature. Interestingly, the combined average weighted UniFrac distances for HCs were significantly lower (*p*<0.05) than the combined average weighted UniFrac distances for IBS subjects. This significant decrease was not observed in the unweighted analysis, indicating that HC samples may be more stable than IBS samples during storage based on the relative abundances of specific OTUs.

### III. Stability of Bacterial Groups During Storage

**Figure 3:**
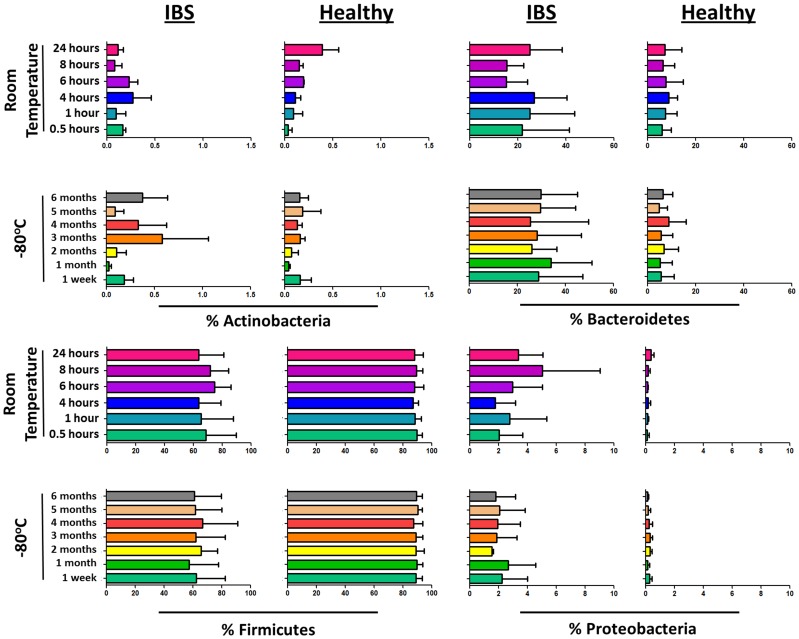
Abundances of dominant phyla in samples. Abundances (% of total 16S rRNA sequences) of the predominant bacterial phyla in healthy control and IBS patient fecal sample aliquots exposed to room temperature and −80°C for different lengths of time.

The percentages of bacterial taxa displayed a non-normal distribution that was likely due the presence of zero values in the data set. All data sets exhibited a normal distribution following log_10_ transformation. Although we observed a significant separation of the composition of the microbiota between samples stored at room temperature and −80°C in one IBS individual (IBS1), we were unable to identify any specific OTUs that were significantly different in concentration between the storage temperatures in any of the four individuals investigated (Table 1). Additionally, the abundance of different bacterial taxa identified by OTU sequence alignments were not found to significantly fluctuate in IBS patients and HC over time during storage at either room temperature or −80°C. Figure 3 displays the variation of dominant phyla in fecal samples during storage. The Bacteroidetes and Proteobacteria phyla exhibited higher concentrations in IBS patients (Figure 3). It has been previously reported by our research group that the Proteobacteria phylum and Gammaproteobacteria class are significantly elevated in IBS patients [14]; however, the Bacteroidetes phylum was not found to be associated with IBS.

**Table 1:** OTUs most significantly altered between room temperature and −80°C storage for all samples and each individual.

Sample Origin	OTU ID	Consensus Lineage	*p* value	Bonferronicorrected*p* value	RT mean%	−80°Cmean %
**All Samples**	5780	Firmicutes;Clostridia;Clostridiales;Lachnospiraceae	0.006	5.951	1.59×10^−04^	2.44×10^−05^
	5434	Firmicutes;Clostridia;Clostridiales;Lachnospiraceae	0.010	9.992	1.11×10^−04^	2.55×10^−05^
	4167	Firmicutes;Clostridia;Clostridiales	0.014	14.530	1.86×10^−04^	2.52×10^−05^
	1710	Bacteroidetes;Bacteroidia;Bacteroidales;Rikenellaceae;Alistipes	0.029	29.303	2.72×10^−04^	7.03×10^−05^
	8336	Firmicutes;Clostridia;Clostridiales;Lachnospiraceae	0.032	32.433	2.48×10^−04^	5.12×10^−05^
	135	Bacteroidetes;Bacteroidia;Bacteroidales;Bacteroidaceae;Bacteroides	0.033	33.398	1.73×10^−05^	9.33×10^−05^
	3326	Firmicutes;Clostridia;Clostridiales;Ruminococcaceae;Faecalibacterium	0.035	35.958	1.79×10^−04^	5.49×10^−05^
	6032	Bacteria	0.038	38.225	1.16×10^−04^	2.70×10_−05_
	7971	Firmicutes;Clostridia;Clostridiales;Ruminococcaceae;Faecalibacterium	0.039	39.843	2.55×10^−05^	1.96×10^−04^
	142	Firmicutes;Clostridia;Clostridiales;Incertae Sedis XIV;Blautia	0.042	42.308	1.89×10^−03^	2.85×10^−03^
**IBS 1**	2064	Bacteroidetes;Bacteroidia;Bacteroidales;Prevotellaceae;Prevotella	0.003	0.387	4.67×10^−04^	1.94×10^−03^
	6839	Firmicutes;Clostridia;Clostridiales	0.006	0.799	7.61×10^−03^	3.18×10^−03^
	7540	Firmicutes;Clostridia;Clostridiales;Lachnospiraceae	0.027	3.486	1.57×10^−02^	9.06×10^−03^
	8513	Firmicutes;Clostridia;Clostridiales;Lachnospiraceae	0.031	3.996	1.82×10^−02^	1.52×10^−02^
	7603	Bacteroidetes;Bacteroidia;Bacteroidales;Prevotellaceae;Prevotella	0.042	5.304	8.87×10^−04^	2.25×10^−03^
	6717	Firmicutes;Clostridia;Clostridiales	0.051	6.425	8.86×10^−04^	4.02×10^−04^
	2944	Bacteroidetes;Bacteroidia;Bacteroidales;Bacteroidaceae;Bacteroides	0.051	6.483	1.02×10^−02^	6.57×10^−03^
	3846	Bacteroidetes;Bacteroidia;Bacteroidales;Bacteroidaceae;Bacteroides	0.058	7.373	1.04×10^−03^	4.19×10^−04^
	6927	ProteoGammaproteoEnterobacteriales;Enterobacteriaceae;Escherichia/Shigella	0.060	7.682	6.64×10^−03^	2.16×10_−03_
	8068	Firmicutes;Clostridia;Clostridiales	0.061	7.693	1.57×10_−03_	7.12×10^−04^
**IBS 2**	5681	Firmicutes;Clostridia;Clostridiales;Eubacteriaceae;Eubacterium	0.002	0.723	4.49×10^−04^	1.46×10^−03^
	5276	Firmicutes;Clostridia;Clostridiales;Lachnospiraceae;Dorea	0.003	0.815	1.47×10^−04^	5.22×10^−04^
	6589	Firmicutes;Clostridia;Clostridiales;Ruminococcaceae;Oscillibacter	0.006	1.912	2.80×10^−02^	1.43×10^−02^
	1971	Firmicutes;Clostridia;Clostridiales;Ruminococcaceae;Faecalibacterium	0.006	1.929	1.14×10^−02^	5.07×10^−03^
	6815	Bacteria	0.009	2.685	2.82×10^−04^	8.56×10^−04^
	6631	Firmicutes;Clostridia;Clostridiales;Ruminococcaceae;Faecalibacterium	0.011	3.388	1.21×10^−03^	3.98×10^−04^
	343	Firmicutes;Clostridia;Clostridiales;Lachnospiraceae	0.013	3.829	2.87×10^−04^	8.79×10^−04^
	8906	Firmicutes;Clostridia;Clostridiales;Lachnospiraceae	0.014	4.109	2.25×10^−04^	7.60×10^−04^
	9044	Firmicutes;Clostridia;Clostridiales	0.017	5.211	9.61×10^−04^	3.06×10^−03^
	6399	Firmicutes;Clostridia;Clostridiales;Incertae Sedis XIV;Blautia	0.022	6.721	7.31×10^−04^	1.29×10^−03^
**HC 1**	452	Firmicutes;Clostridia;Clostridiales;Lachnospiraceae	0.003	0.849	1.57×10^−03^	9.82×10^−04^
	3634	Firmicutes;Clostridia;Clostridiales;Lachnospiraceae	0.005	1.636	5.67×10^−04^	1.95×10^−04^
	3163	Firmicutes;Clostridia;Clostridiales;Incertae Sedis XIV;Blautia	0.013	4.101	5.69×10^−03^	3.27×10^−03^
	809	Firmicutes;Clostridia;Clostridiales;Lachnospiraceae;Roseburia	0.014	4.507	2.36×10^−04^	5.49×10^−04^
	9486	ProteoBetaproteoBurkholderiales;Alcaligenaceae;Parasutterella	0.015	4.755	9.26×10^−04^	1.70×10^−03^
	6663	Firmicutes;Clostridia;Clostridiales;Ruminococcaceae;Faecalibacterium	0.024	7.418	3.50×10^−04^	1.53×10^−04^
	4191	Bacteria	0.026	8.003	1.70×10^−03^	8.91×10^−04^
	6951	Firmicutes;Clostridia;Clostridiales;Ruminococcaceae;Faecalibacterium	0.031	9.599	5.31×10^−04^	2.58×10^−04^
	3893	Firmicutes;Clostridia;Clostridiales	0.033	10.175	8.86×10^−04^	1.68×10^−03^
	7567	Firmicutes;Clostridia;Clostridiales;Incertae Sedis XIV;Blautia	0.039	12.077	4.77×10^−04^	1.06×10^−03^
**HC 2**	5281	Firmicutes;Clostridia;Clostridiales;Ruminococcaceae	0.001	0.259	5.01×10^−04^	1.63×10^−03^
	1331	Firmicutes;Clostridia;Clostridiales;Lachnospiraceae	0.004	1.039	1.89×10^−04^	1.27×10^−03^
	379	Firmicutes;Clostridia;Clostridiales;Ruminococcaceae;Faecalibacterium	0.006	1.291	2.03×10^−03^	8.78×10^−04^
	8605	Firmicutes;Clostridia;Clostridiales	0.008	1.880	4.71×10^−04^	1.41×10^−03^
	142	Firmicutes;Clostridia;Clostridiales;Incertae Sedis XIV;Blautia	0.017	3.977	2.92×10^−03^	4.81×10^−03^
	3095	Firmicutes;Clostridia;Clostridiales	0.019	4.397	1.68×10^−03^	3.02×10^−03^
	331	Firmicutes	0.023	5.269	6.00×10^−04^	1.46×10^−03^
	2380	Firmicutes;Clostridia;Clostridiales;Ruminococcaceae;Faecalibacterium	0.023	5.348	1.41×10^−03^	6.28×10^−04^
	9034	Firmicutes;Clostridia;Clostridiales;Ruminococcaceae;Faecalibacterium	0.024	5.489	5.86×10^−03^	3.71×10^−03^
	9044	Firmicutes;Clostridia;Clostridiales	0.024	5.659	4.21×10^−03^	6.87×10^−03^

## Discussion

Until the advent of DNA-based methods to characterize complex bacterial communities in biological samples, it was widely accepted that human fecal samples must be immediately frozen upon collection in order to preserve the composition of the microbiota [26]. This assumption proved practically difficult in human clinical trials, where collected fecal samples could be exposed to room temperature for hours before reaching the laboratory. Additionally, depending on the speed of subject recruitment, fecal samples may have to be stored in deep freezing conditions for long periods of time before isolating DNA.

A recent study using high throughput pyrosequencing of the 16S rRNA gene revealed that the phylogenetic structure of the microbiota did not significantly differ between three- and fourteen-day old fecal samples stored at a range of temperatures (20, 4, −20 and −80°C) [10]. However, Lauber *et al*’s [10] study lacked a baseline sample representing the structure of the fecal microbiota immediately following defecation. Thus, it is possible that the microbiota in fecal samples degraded rapidly and maintained this composition over time. To address these points we designed a study where a baseline aliquot (30 minutes following defecation) was collected and compared to aliquots of the same sample stored at room temperature over a twenty four hour period. We also investigated the stability of the fecal microbiota from aliquots of the same samples stored at −80°C over six months. Furthermore, as fecal samples from ulcerative colitis and IBS patients have significantly higher protease activity [27], it is possible that the fecal microbiota from individuals with intestinal disease may be affected differently by the length of exposure to room or freezing temperatures. Thus, healthy individuals and IBS patients were included in this study.

When directly comparing the composition of the intestinal microbiota from all subjects and samples stored at room temperature using UniFrac distances[22]–[24], we found that samples segregated based on the subject they originated from rather than the length of time they were exposed to room temperature. This finding suggests that fecal samples kept at room temperature for up to twenty four hours retain a microbiota that is similar in composition to a fresh sample from that individual. Likewise, during long-term storage at −80°C, we found the composition of the microbiota from all subjects and samples segregated based on the subject they originated from rather than the length of time they were exposed to deep freezing conditions. This finding suggests that fecal samples kept at −80°C for up to six months also retain a microbiota that is similar in composition to a fresh sample from that individual. However, we found that the composition of the microbiota from samples stored at room temperature significantly segregated from the microbiota of samples stored at −80°C for one IBS individual. The samples from the second IBS individual did not display the same significant separation between storage temperatures; however clustering of samples on a weighted PCoA displayed greater variation when compared to HC, implying that the microbiota from this IBS patient may also be less stable at room temperature to that of healthy controls. Indeed, the microbiota from both HC samples exhibited lower average weighted UniFrac values than the microbiota from both IBS samples. In support of this observation, our weighted PCoA analysis shows that HC samples cluster tighter than IBS samples. This observation suggests that abundant taxa in the HC samples in our study are less affected by temperature than the IBS analyzed in our study. Although, the IBS samples we analyzed were more affected by storage temperature than HC, the microbiota in these samples bore more similarity to their individual of origin than to storage temperature, and we did not identify any specific OTU or higher taxonomic group that significantly differed between storage temperatures in IBS individuals. This indicates that although IBS samples stored at room temperature for up to 24 hours may alter in microbial composition, these samples still closely represent the intestinal microbiota of that individual. It is unclear why the microbiota of IBS fecal samples are less stable during storage at room temperature, however as intestinal metabolites are altered in IBS patients [27], [28] it is tempting to speculate that elevated levels of proteases and acids may result in faster degradation of fecal DNA.

Our study reports a relatively stable microbiota in fecal samples stored at room temperature or −80°C over time, however we do not see identical bacterial compositions in all samples from the same host. It has been reported that the length of time at different storage temperatures affects the densities of specific bacterial groups [29] in swine feces. Although we did not see a significant effect of length of time at a storage temperature on bacterial taxonomic groups or individual species-level OTUs, our analysis did not provide the resolution to investigate fluctuations of taxa at the species or strain level. Additionally, we did not investigate the stability of the microbiota in fecal samples between 8 and 24 hours at room temperature, where the microbiota may be less stable. It is also important to note that although we generated a relative large number of sequences per sample, our depth of coverage will not detect very low abundant taxa. It is, therefore, possible that low abundance taxa may be affected by storage temperature. Furthermore, fecal DNA used in our study to characterize the microbiota of fecal samples was stored at −80°C for different lengths of time, and we have not investigated that stability of fecal DNA over time during freezing conditions.

In conclusion we report that fecal samples exposed to room or deep freezing temperatures for up to twenty four hours and six months, respectively, exhibit a microbial composition and diversity that shares more identity with its host of origin than any other sample. This study validates the approach of storing samples at −80°C until a full complement of fecal samples is acquired in order to investigate the composition of the fecal microbiota in healthy controls and IBS patients.
